# New horizon for high performance Mg-based biomaterial with uniform degradation behavior: Formation of stacking faults

**DOI:** 10.1038/srep13933

**Published:** 2015-09-09

**Authors:** Jinghuai Zhang, Chi Xu, Yongbin Jing, Shuhui Lv, Shujuan Liu, Daqing Fang, Jinpeng Zhuang, Milin Zhang, Ruizhi Wu

**Affiliations:** 1 Key Laboratory of Superlight Materials & Surface Technology, Ministry of Education, College of Material Science and Chemical Engineering, Harbin Engineering University, Harbin 150001, China; 2 Department of orthopedics, The Second Affiliated Hospital of Harbin Medical University, Harbin 150001, China; 3 School of Materials Science and Engineering, Changchun University of Science and Technology, Changchun 130022, China; 4 Department of Materials Physics and Chemistry, Harbin Institute of Technology, Harbin 150001, China; 5 School of Materials Science and Engineering, Taiyuan University of Science and Technology, Taiyuan, 030024, China; 6 The Key Laboratory of Myocardial Ischemia, Harbin Medical University, Ministry of Education, Heilongjiang Province, China

## Abstract

Designing the new microstructure is an effective way to accelerate the biomedical application of magnesium (Mg) alloys. In this study, a novel Mg–8Er–1Zn alloy with profuse nano-spaced basal plane stacking faults (SFs) was prepared by combined processes of direct-chill semi-continuous casting, heat-treatment and hot-extrusion. The formation of SFs made the alloy possess outstanding comprehensive performance as the biodegradable implant material. The ultimate tensile strength (UTS: 318 MPa), tensile yield strength (TYS: 207 MPa) and elongation (21%) of the alloy with SFs were superior to those of most reported degradable Mg-based alloys. This new alloy showed acceptable biotoxicity and degradation rate (0.34 mm/year), and the latter could be further slowed down through optimizing the microstructure. Most amazing of all, the uniquely uniform *in vitro*/*vivo* corrosion behavior was obtained due to the formation of SFs. Accordingly we proposed an original corrosion mechanism for the novel Mg alloy with SFs. The present study opens a new horizon for developing new Mg-based biomaterials with highly desirable performances.

Magnesium (Mg) ion is the fourth most abundant cation in human body and an essential element for many biochemical functions in the living processes of human body^1^. As one of the essential elements, Mg has outstanding biocompatibility. Mg alloys would degrade in the physiological environment, and the dissolved Mg ions have stimulatory effects on the growth of new bone tissue^2^,^3^,^4^,^5^,^6^,^7^,^8^. Meanwhile, the density, strength and elastic modulus of Mg alloys are close to natural bones compared with other medical implant materials^9^. All of these show great potential of Mg to be used for degradable biomaterials^2^,^10^. However, Mg alloys also have some issues to serve as biodegradable implants. The most notable one is the degradation behavior, i.e. nonuniform degradation mode and too fast degradation rate^11^,^12^,^13^,^14^, which is the key problem of restricting the application of degradable Mg-based alloys^15^,^16^. It is expected to overcome disadvantages of degradable Mg-based alloy and retain its advantages simultaneously, though it seems not easy to achieve.

As we known, the microstructure characteristic is the key factor to determine material performances, and it is the same to mechanical properties and corrosion resistance of Mg alloys^17^. The classical strengthening methods of Mg alloys mainly include the following ones^18^. (1) Solution strengthening. Addition of alloying elements to Mg for forming solid solution is an easy schedule of reinforcement. As alloying elements of degradable Mg-based alloys, several elements have been proved to be non-toxic to some extent, such as Ca^10^, Zn^19^,^20^,^21^, Zr^22^, Sr^23^, Li^24^, part of REs^25^,^26^, etc. Single-phase solid solution Mg alloys are considered to be comparatively ideal degradable Mg-based materials due to the relatively uniform microstructure by some researchers^19^,^27^,^28^. However, solution strengthening is a relatively less effective reinforcement way in Mg alloys^29^. In addition, the segregation of alloying elements may also be formed^30^, and the presence of inhomogeneities would inevitably cause local corrosion due to the formation of galvanic cells. (2) Secondary phase strengthening (Dispersion strengthening or precipitation strengthening). Many studies have demonstrated that the strength of Mg alloys can be significantly promoted by forming secondary phase particles^28^,^31^,^32^,^33^. Therefore, high-strength Mg alloys usually contain amounts of intermetallic particles. However, it should also be noted that the majority of secondary phase particles enhance the strength at the expense of ductility^32^,^34^. More seriously, micro-galvanic coupling would build up between the cathodic secondary phase particle and the anodic Mg matrix, and consequently severe pitting corrosion would occur around the particles^35^,^36^. (3) Grain refinement strengthening (Grain boundary strengthening). Generally speaking, grain refinement strengthening can promote strength and plasticity simultaneously. Meanwhile most of researchers considered that grain refinement can enhance corrosion resistance of Mg alloys^37^,^38^, while sometimes the preferred corrosion of grain boundary is also not ignored^39^,^40^.

In view of shortcomings of the common Mg alloys as biodegradable implants, recently designing new uniform microstructure for degradable Mg-based materials become another point of view. The typically experimental alloys are Mg alloys with long period stacking ordered (LPSO) structure. It has been well confirmed that LPSO phases play an important role in improving mechanical properties of Mg alloys as structure materials due to coherent LPSO phase-Mg matrix interface, formation of kink bands, etc.^41^,^42^. More recently, the effect of LPSO phase on bio-corrosion properties starts to attract attention. Zhang *et al.*^12^ reported that as-cast Mg–5Gd–1Zn–0.6Zr (wt.%) alloy with LPSO phase located near grain boundaries exhibited slower corrosion rate and relatively uniform corrosion morphology compared with the alloy without LPSO structure. Peng *et al.*^43^ investigated the effect of 18R and 14H LPSO phases on degradation behavior, and the results demonstrated that 14H LPSO phase in the grain interior was more effective to improve corrosion resistance than 18R LPSO phase distributed along grain boundaries. The above researches showed the feasibility of developing new degradable Mg-based alloys by introducing special structure.

During the study of LPSO structure, a few researchers also found stacking faults (SFs) in as-cast^41^ or heat-treated^44^ Mg–RE–Zn alloys. Unfortunately, in these studies, SFs were just a small part of the microstructure rather than the main structural morphology, and also did not draw enough attention. In this work, we use traditional preparation method but specific process to fabricate the Mg–8Er–1Zn (wt.%) alloy, and encouragingly we achieve the microstructure which was just composed of SFs and Mg matrix. Even more excitingly, the uniform and nano-spaced SFs provide outstanding performance to the degradable Mg-based alloy, especially the uniform degradation behavior. We consider that the development of the alloy with profuse SFs is a quite effective way to solve the main issues existed in the present degradable Mg-based alloys, and opens up a new horizon in the area of degradable Mg-based.

## Results

### Microstructure: SFs formed in Mg–8Er–1Zn alloy

**Figure 1:**
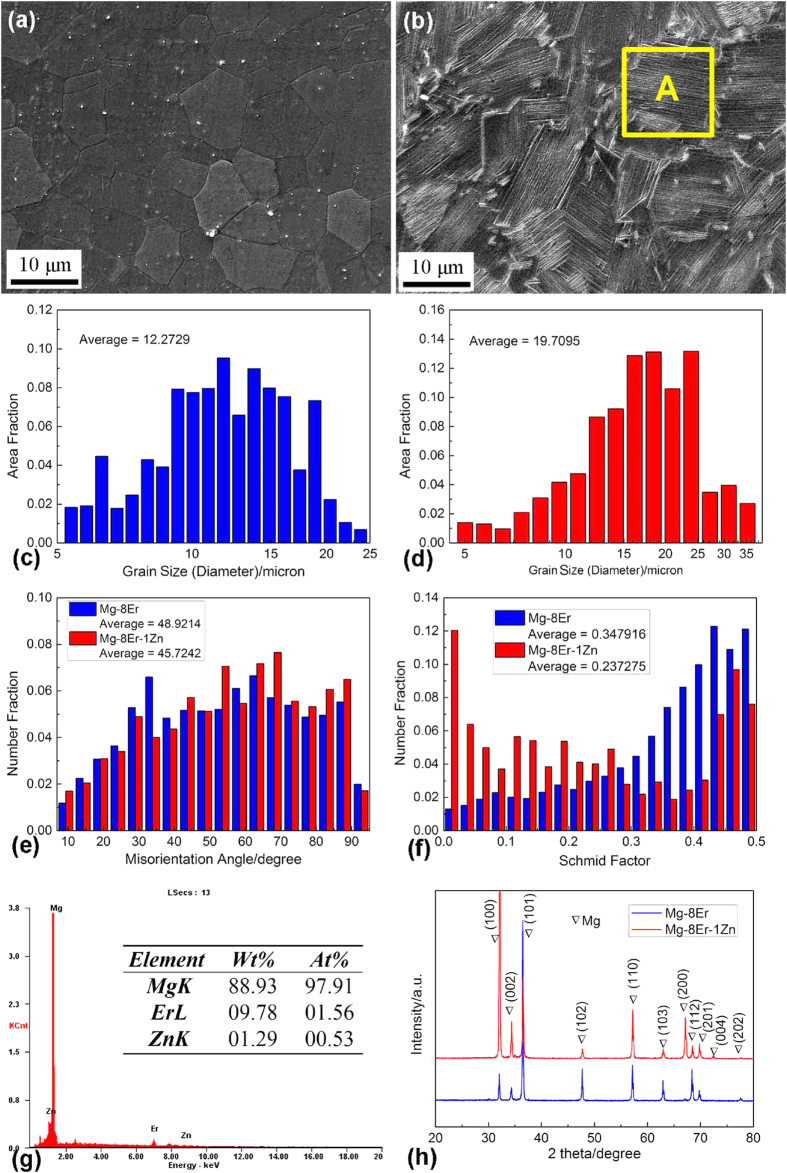
Microstructure analyses of tested alloys. SEM images of (**a**) Mg–8Er and (**b**) Mg–8Er–1Zn; grain size distributions of (**c**) Mg–8Er and (**d**) Mg–8Er–1Zn; (**e**) misorientation angle distributions of the two alloys; (**f**) (0001) <
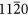
> Schmid factor distributions along the ED for the two alloys; (**g**) EDS result of area A in (**b**); (**h**) XRD patterns of the two alloys.

Figure 1a shows the SEM image of Mg–8Er reference alloy. It can be seen that the microstructure of the binary alloy contains primary α-Mg grains and a few of secondary phase particles. Surprisingly, with adding Zn in small amounts (~1 wt.%), the alloy microstructure changes significantly. Not more particles but additional profuse lamellae are formed and distributed throughout the whole Mg matrix with a specific orientation in the same α-Mg grain. In addition, some other information is obtained from EBSD analyses. The average grain size of Mg–8Er–1Zn alloy is larger than that of Mg–8Er alloy (Fig. 1c,d). Both the two alloys are mainly composed of large angle grain boundaries and their number fractions are roughly the same (see Fig. 1e). It is much different between the fraction distributions of Schmid factor for basal slip along the extrusion direction (ED) of the two alloys, as show in Fig. 1f. The basal slip Schmid factors of Mg–8Er alloy tend to high values and its average value is about 0.35, while the average basal slip Schmid factors of Mg–4Er–4Gd–1Zn alloy with SFs is just about 0.24. The change of Schmid factor would affect the mechanical properties of the alloys. The EDS analysis in SEM mode (Fig. 1f) just estimates that the lamellae region is slightly rich in Er and Zn elements, thus further analyses about the lamellae are required. Figure 1g shows the XRD patterns of Mg–8Er and Mg–8Er–1Zn alloys. Interestingly, all the peaks are indexed into α-Mg phase in both the two alloys, and no other phases can be detected.

**Figure 2:**
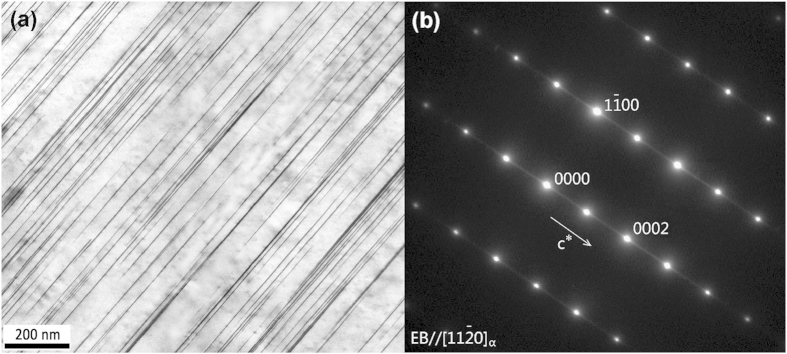
TEM image (a) and SAED pattern (b) of SFs in Mg–8Er–1Zn alloy.

To further confirm the lamellae formed in Mg–8Er–1Zn alloy, the morphology and structure were characterized by TEM analyses. As shown in Fig. 2a, the space between the parallel lamellae ranges from several nanometers to 100 nanometers. In the corresponding selected area electron diffraction (SAED) pattern (Fig. 2b), just a 2H-Mg crystal structure is identified, and no periodic extra spots but streaking between the diffraction spots along c-axis confirm the formation of basal plane stacking faults (SFs) instead of LPSO structure^43^. The above observation and analyses demonstrate that a novel microstructure of Mg alloy is obtained, in which the nano-spaced basal plane SFs are uniform in distribution and have different orientations in different α-Mg grains.

### Mechanical properties of the alloy with SFs

**Figure 3:**
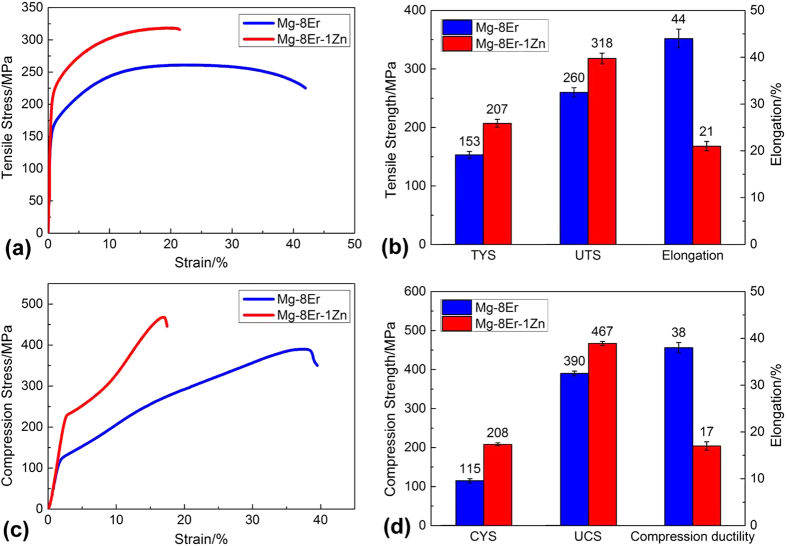
Mechanical properties of the alloys. (**a**) Tensile stress-strain curves; (**b**) tensile properties; (**c**) compression stress-strain curves; (**d**) compression properties.

The mechanical properties of the Mg–8Er–1Zn alloy and reference Mg–8Er alloy are characterized by tensile tests and compressive tests. Figure 3a,b show the tensile properties. As expected, the as-extruded Mg–8Er alloy exhibits low tensile strength, making itself not suitable for biomedical application. Surprisingly, when a little amount of Zn (~1 wt.%) is added in the binary alloy, the tensile strength is significantly enhanced though the elongation is less than that of the Mg–8Er binary alloy. The tensile yield strength (TYS), ultimate tensile strength (UTS) and elongation for Mg–8Er–1Zn alloy are 207 MPa, 318 MPa and 21%, respectively. On the other hand, as shown in Fig. 3c and d, Mg–8Er–1Zn alloy also exhibits the high compression yield strength (CYS: 208 MPa), ultimate compression strength (UCS: 467 MPa) and enough compression ductility (17%). The results indicate that the strength and ductility of Mg–8Er–1Zn satisfy the demands of biodegradable implant material^45^.

### Electrochemical corrosion behavior of the alloy with SFs

**Figure 4:**
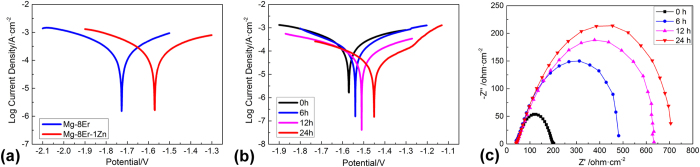
Polarization curves and Nyquist plots of the alloys. (**a**) Polarization curves of Mg–8Er alloy and Mg–8Er–1Zn alloy; (**b**) polarization curves and (**c**) Nyquist plots of Mg–8Er–1Zn alloy with different immersion times in SBF.

Potentiodynamic polarization tests were carried out to estimate the corrosion resistance of the alloys. As shown in Fig. 4a, Mg–8Er–1Zn exhibits higher corrosion potential and lower corrosion current density compared with Mg–8Er alloy. Figure 4b shows the effect of immersion times on corrosion resistance of Mg–8Er–1Zn alloy using electrochemical polarization measurement. There is an obvious trend that the corrosion potential moves to the positive direction and the corrosion current density decreases with increasing immersion time. These dynamic changes demonstrate the gradual improvement of corrosion resistance, and imply that a stable corrosion product layer would be gradually formed on the surface of Mg–8Er–1Zn alloy in SBF. The electrochemical impedance spectroscopy (EIS) was also employed to investigate the changes on the surface of Mg–8Er–1Zn alloy in SBF solution after different immersion times up to 24 h, as shown in Fig. 4c in the form of a Nyquist plot measured at corrosion potential. It can be seen that all the curves have a single capacitive loop at all frequencies and the inductance loop is hardly detected. These EIS spectra are similar except the increasing diameter with increasing immersing time. The diameter of the capacitive semi-circle is closely related to the degradation rate, and the increasing diameter indicates the formation of gradually more effective anti-corrosion film and the lower degradation rate, which is consistent with the electrochemical polarization measurement.

### Corrosion rate of the alloy with SFs in SBF

Table 1 shows a comparison of the corrosion rates in the same units (mm/y) by the three techniques of weight loss, hydrogen evolution and Tafel extrapolation of polarization curves. It can be seen that there is good agreement between the low corrosion rates evaluated from the hydrogen evolution rate and that evaluated from the weight loss rate. However, there is a large deviation for the corrosion rate determined from Tafel extrapolation compared with the weight loss rate, which is in agreement with the observation of the review of Atrens^46^. In this work, the corrosion rates from H_2_ collection and weight loss is the average value of long-term tests (10 days), while the corrosion rate from polarization curves is the value of short-term tests (after 10 min immersion). Therefore, the time is one of the effect factors, which is supported by Fig. 4b,c. Erinc *et al.*^40^ has suggested the performance criterions for biodegradable Mg alloys and among them, the corrosion rate in SBF should be less than 0.5 mm/year. It is important to note that the low corrosion rate of Mg–8Er–1Zn alloy with SFs in SBF meets the performance criterion of Mg alloys as biomaterials. Furthermore, we have preliminarily found that the further improved corrosion resistance of Mg–RE–Zn alloy with SFs can be achieved, without sacrificing other performance, through optimizing the distribution of SFs and refining the grain size.

### *In vitro* corrosion film of the alloy with SFs

**Figure 5:**
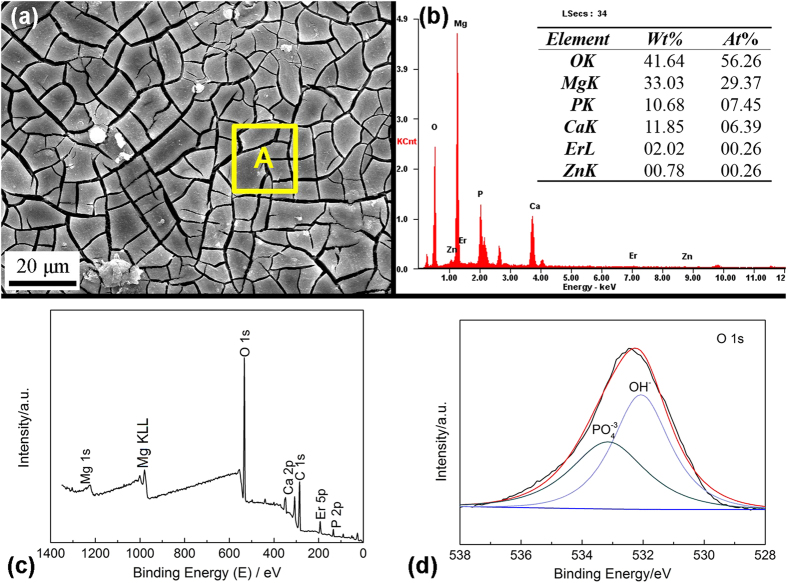
Morphology and composition of the corrosion layer of Mg–8Er–1Zn alloy immersed in SBF for 10 days. (**a**) SEM image of the corrosion layer; (**b**) EDS result of area A in (**a**); (**c**) XPS spectrum of the corrosion layer; (**d**) high resolution XPS spectrum of O 1 s.

Corrosion layer also plays an important role in determining the corrosion resistance of Mg alloys^47^. The long period immersion test for Mg–8Er–1Zn samples was carried out in SBF for 10 days, and the morphology of the corrosion layer is shown in Fig. 5a. A smooth and compact film can be observed on the surface of Mg–8Er–1Zn alloy, with some cracks caused by dehydration of the layer during the drying process and under the vacuum of the SEM chamber. According to the result of EDS analysis in Fig. 5b, the product layer contained high contents of P and Ca except Mg and O, and it implies the mixture of hydroxides and hydroxyapatite. To define the corrosion film composition of Mg–8Er–1Zn, XPS analysis has been carried out, and the signals of Mg, O, Ca, P and Er are observed in Fig. 5a. It is known that the position of the O 1 s peak observed at 531.2 eV and 533.5 eV are corresponding to the OH^−^ and PO_4_^3−^, respectively (Fig. 5b)^47^. In addition, it can confirm the element P exists in the film in a form of PO_4_^3−^ from the binding energies of P 2p and Ca 2p. Thus, Mg(OH)_2_, Ca_5_(PO_4_)_3_(OH) and Er(OH)_3_ are confirmed in the corrosion product film from above results. It is considered that high content Er^3+^ in the hydroxide film structure can increase the local positive charge and inhibit the penetration of detrimental anions (Cl^−^) across the corrosion film by trapping anions, thus inhibit further corrosion^48^. On the other hand, Ca_5_(PO_4_)_3_(OH) as the an essential component of bone, is beneficial for alloy served as biodegradable material within bone.

### *In vitro* corrosion mode of the alloy with SFs

**Figure 6:**
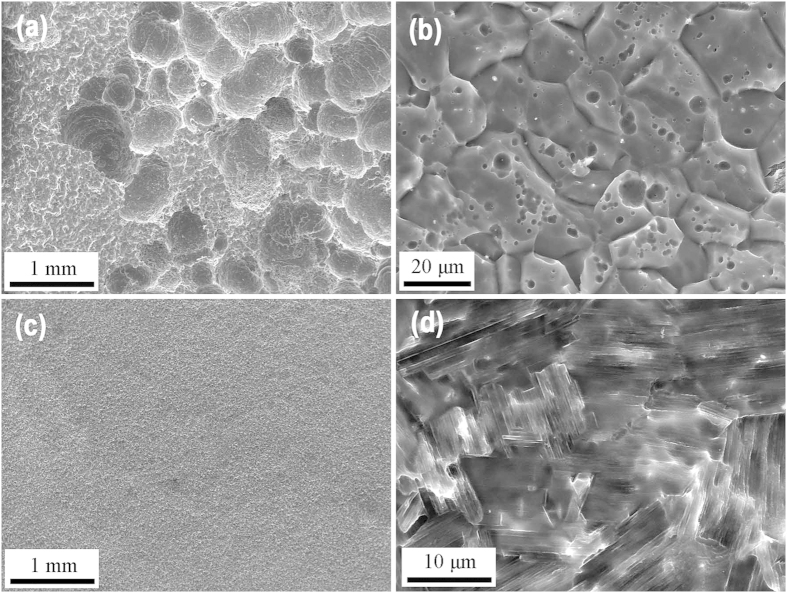
SEM images showing the surface morphologies of the alloys after immersion in SBF solution at 37 °C for 10 days. (**a**,**b**) Mg–8Er; (**c**,**d**) Mg–8Er–1Zn.

Uniform corrosion behavior, in other word, controlled corrosion rates, is particularly important for degradable Mg-based implant to extend their load-bearing period. Figure 6 shows the corrosion morphologies of Mg–8Er and Mg–8Er–1Zn alloys immersed in SBF for 10 days after removing the corrosion products. For Mg–8Er alloy (Fig. 6a,b), the severe localized corrosion pits are clearly visible on the surface. Compared Mg–8Er alloy, it is indeed encouraging to note that our new designed Mg–8Er–1Zn alloy with profuse SFs exhibits highly desirable uniform corrosion morphology (Fig. 6c,d).

### *In vivo* corrosion behavior of the alloy with SFs

**Figure 7:**
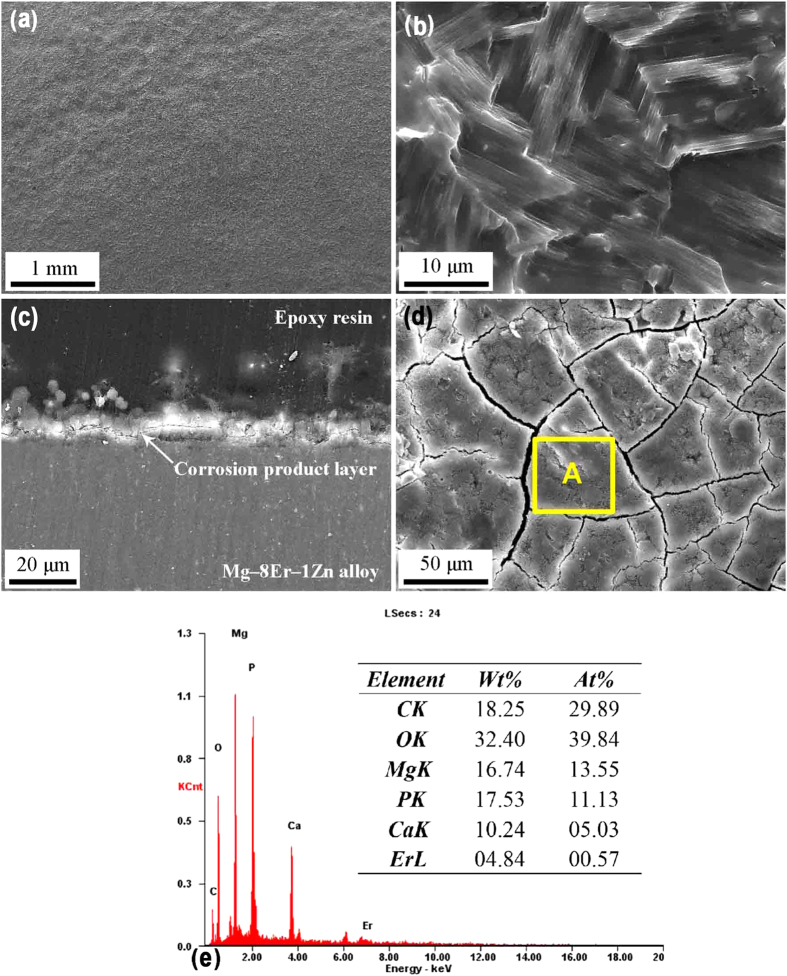
*In vivo* corrosion morphologies and product layer of the alloys after implanted in erector spinae of rabbits for 10 days. (**a**,**b**) Corrosion morphology of Mg–8Er–1Zn alloy; (**c**) cross profile of corrosion layer for Mg–8Er–1Zn alloy; (**d**) corrosion layer of Mg–8Er–1Zn alloy; (**e**) EDS result of area A in (**d**).

Witte *et al.*^49^ comparatively studied the corrosion rates of AZ91D and LAE442 alloys under standard *in vitro* environmental conditions and the corrosion rates *in vivo* animal model. They found that *in vivo* corrosion was lower than *in vitro* corrosion of the two alloys, and furthermore, the tendency of the corrosion rates obtained from *in vitro* corrosion tests was in the opposite direction as those obtained from the *in vivo* study. Their results suggested that the conclusion drawn from *in vitro* corrosion tests may not be sure to predict *in vivo* corrosion behavior very well. Therefore, *in vivo* corrosion of Mg–8Er–1Zn alloy was also initially tested on rabbits. Figure 7 shows the surface morphology of Mg–8Er–1Zn alloy sample after implanted in erector spinae of rabbits for 10 days. It reveals that uniform corrosion feature is observed clearly again whether from the low or high magnified images (Fig. 7a,b), which is the same as *in vitro* corrosion morphology for 10 days (Fig. 6c,d). The cross section of Mg–8Er–1Zn alloy sample appears straight, as shown in Fig. 7c, which further indicates the uniform corrosion mode. Figure 7d shows the corrosion surface film and Fig. 7e gives its main compositions detected by EDS. The results indicate the corrosion film is roughly the same as that formed in SBF.

### Cytotoxicity of the alloy with SFs

**Figure 8:**
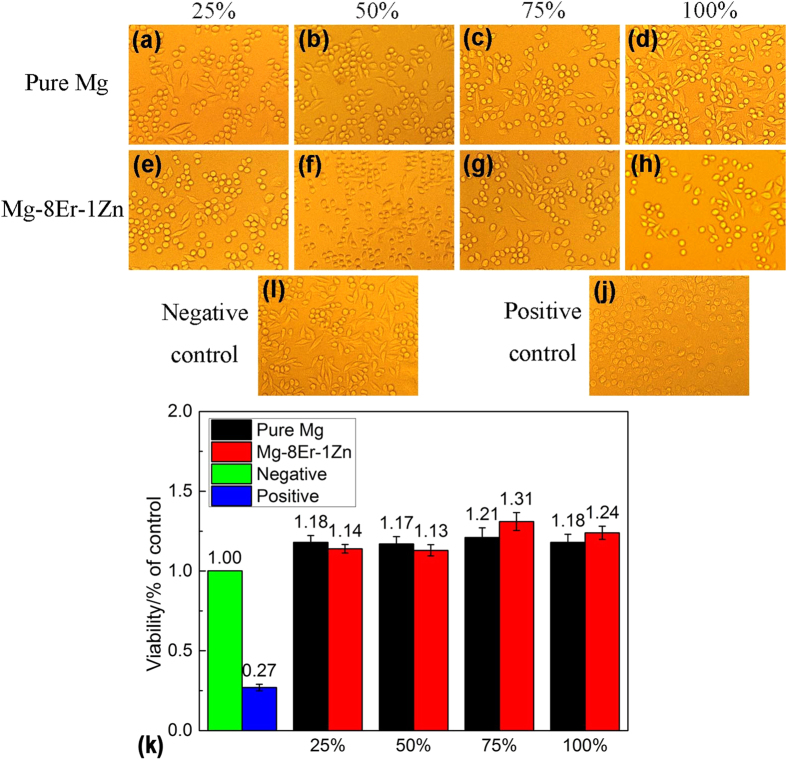
Optical morphologies (×200) and cell viability of L-929 cells after cultured at 25%, 50%, 75%, 100% extracts of Mg–8Er–1Zn alloy for 1 day, and compared with pure Mg, blank negative control and positive control groups. (**a**–**d**) Optical morphologies of pure Mg and (**e**–**h**) optical morphologies of Mg–8Er–1Zn alloy; (**i**) optical morphology of blank negative control group; (**j**) optical morphology of positive control group; (**k**) cell viability.

Acceptable biocompatibility is the key property to a biomaterial, thus the comparative cytocompatibility tests were carried out to evaluate the biocompatibility of new designed Mg–8Er–1Zn alloy. Pure Mg acted as reference due to its accepted cytotoxicity. Figure 8a to j shows the L-929 cell morphologies cultured in different concentration extracts of pure Mg and Mg–8Er–1Zn alloy for 1 day. The comparative observation indicates all the results for different percentage composition pure Mg and Mg–8Er–1Zn alloy extracts and the negative control exhibit healthy morphology with round and spindle shapes, suggesting the good biocompatibility of Mg–8Er–1Zn alloy. Here, it should be noted that the optical microscope photographs only show the morphology but not the amount of the cells because the photographs were taken at a certain part randomly and not an overall view. As it shown in Fig. 8k, the cell viabilities cultured in Mg–8Er–1Zn alloy extraction medium at different percentage composition are all higher than these cultured in negative control and positive control groups. It should be noticed that the high concentration extracts (75% and 100%) of Mg–8Er–1Zn alloy contribute to higher cell viability than those of pure Mg at the same concentration. Therefore, basic biosafety of Mg–8Er–1Zn alloy is confirmed for biomedical applications.

## Discussion

In this work, we firstly report a novel Mg–Er–Zn alloy with profuse basal plane SFs, and the alloy exhibits not only low bio–corrosion rate but also uniform corrosion mode in the premise of high mechanical properties.

### Formation of SFs

Many researches have demonstrated the LPSO structure can be formed in Mg–RE–Zn alloys (RE = Y, Gd, Tb, Dy, Ho, Er, Tm)^50^,^51^ and among them, Mg–Y–Zn, Mg–Gd–Zn and Mg–Dy–Zn alloys were investigated widely. Now we first fabricated a Mg–Er–Zn alloy with profuse basal plane SFs by relatively simple preparation process. According to previous reports^52^, we consider that SFs are the early stage of LPSO structure formation, and the six factors proposed by Ding *et al.*^53^, which promote the formation of LPSO structure, are equally applicable to SFs formation. However, we also propose that the formation conditions of SFs and preparation processes of the alloy with SFs are more rigorous. For example, the more proper ratio of RE and Zn should be required. The intensive study about influence factors of SFs formation in Mg–RE–Zn alloys is underway and the results will be reported elsewhere.

### Influence of SFs on mechanical properties

Compared with vast majority of the previously reported degradable Mg-based alloys, the Mg–8Er–1Zn alloy with SFs exhibits outstanding strength, ductility and tension-compression symmetry^54^. The mechanical properties of Mg–8Er–1Zn alloy, i.e. the yield strength of above 200 MPa and the ductility of about 20%, are acceptable for biomedical applications^40^. Furthermore, it is believed that controlling the grain size and number density of SFs can further improve the mechanical properties.

The formation of SFs is a new and very effective strengthening method for Mg alloys. Jian *et al.*^55^ reported that SFs would interact with dislocations under the applied stress, and consequently the blocking of dislocations by SFs and the cutting of SFs by dislocations could increase the strength. Meanwhile, SFs are also effective in accumulating dislocations, which would enhance strain hardening and contribute to retaining ductility. In addition, according to the EBSD analyses, the high basal slip Schmid factor would be contributed strongly to the high ductility for Mg–8Er alloy; while in contrast, the relatively low basal slip Schmid factor would make some contribution to the high strength for the Mg–8Er–1Zn alloy with SFs.

### Influence of SFs on corrosion behavior

**Figure 9:**
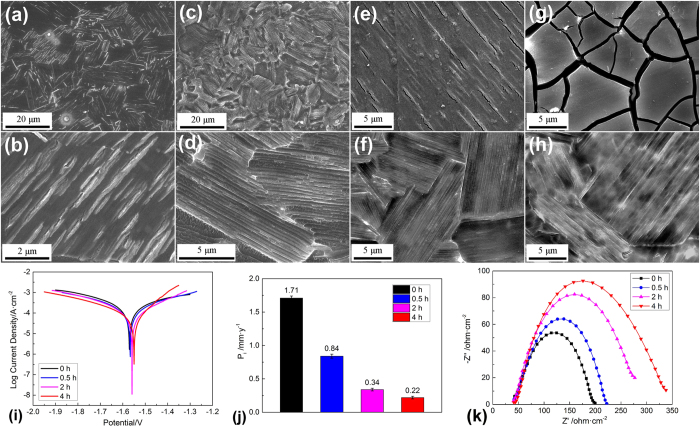
SEM images and electrochemical analyses of Mg–8Er–1Zn alloy after short-term immersion. (**a**,**b**) Corrosion product layer after 0.5 h immersion; (**c**,**d**) corroded surface after 0.5 h immersion; (**e**) corrosion product layer after 2 h immersion; (**f**) corroded surface after 2 h immersion; (**g**) corrosion product layer after 4 h immersion; (**h**) corroded surface after 4 h immersion; (**i**) polarization curves with the corresponding short-term immersion times; (**j**) corrosion rate calculated from polarization curves; (**k**) electrochemical impedance diagrams with the corresponding short-term immersion times.

To figure out the effect of SFs on the corrosion behavior, a series of short period immersion tests were carried out. The changes of typical surface morphology with increasing immersion time are shown in Fig. 9. At the early stage of 0.5 h, large quantities of light strips appear on the product layer under contrast of secondary electron images (Fig. 9a,c), and the cracks (caused by dehydration of the layer during the drying process and under the vacuum of the SEM chamber) along the strips implied a higher density compared with the dark parts of the product layer. With immersion time going on (2 h), the light strips in Fig. 9e became darker, and not that clear as those in Fig. 9c. After immersed for 4 h, strips totally disappear and the product layer turns to be uniform (Fig. 9g). The corroded surfaces are also observed after removing the product layer (Fig. 9b,d,f,h), revealing the special corrosion morphology of Mg–8Er–1Zn alloy with SFs. The long and shallow ditches with a specific orientation in the same α-Mg grain can be observed, especially from Fig. 9d. Furthermore, the corroded surface is flat and homogeneous.

**Figure 10:**
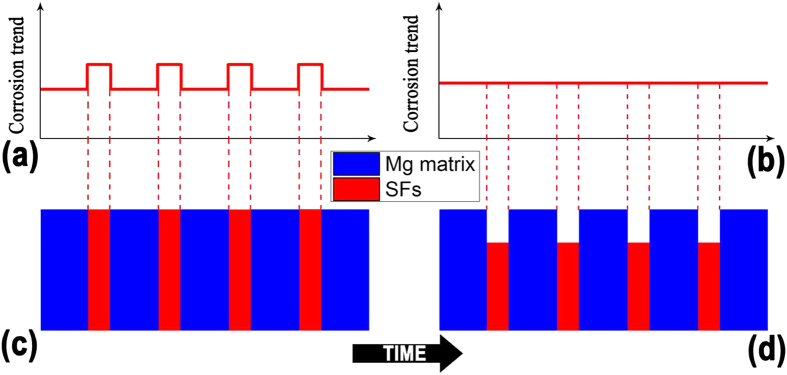
Schematic diagrams of corrosion trend during corrosion process. (**a**) Corrosion trend at start point; (**b**) corrosion trend at a certain time point; (**c**) morphology of alloy with SFs at start point; (**d**) morphology of alloy with SFs at a certain time point.

According to all the above observations and analyses, it is easy to conclude that the basal plane SFs play an important role in forming the uniquely uniform corrosion mode. Based on the special state of SFs in Mg-8Er-1Zn alloy, i.e. special defect structure, one-dimensional nano-morphology and nano-space, Fig. 10 shows a simulation of corrosion trend in Mg alloy with SFs (Mg–8Er–1Zn alloy) during the corrosion process. SFs contain more energy than Mg matrix as defect structure, which contain a higher level of free energy, leading to a higher corrosion probability. Consequently, micro fluctuation of corrosion trend is confirmed on the original bare Mg–8Er–1Zn alloy affected by the distribution of profuse SFs (Fig. 10a). During the corrosion process, SFs are more active and dissolve first as protection of Mg matrix, then the lamellae surface forms (corresponding to Fig. 9d). As corrosion process continues, energy in SFs decreases gradually and reaches a balance level as Mg matrix at a certain time point. Therefore, a horizontal corrosion trend on Mg-8Er-1Zn alloy is formed, as it shown in Fig. 10b. With the same corrosion trend controlled by SFs, a new type of uniform degradation mode is confirmed. It is noted that though SFs contains more energy than Mg matrix, the gap of energy between SFs and Mg matrix is narrowed by negative atoms (as Er and Zn in Mg-8Er-1Zn alloy) gathered on the SFs interface^39^. Therefore, the depth of “ditches” left by dissolved SFs is a small value, which can be confirmed in above experiment. In addition, the corrosion product film rapidly formed on Mg alloy with SFs is also beneficial to corrosion resistance, which is confirmed by the decreasing corrosion rate (Fig. 9j) measured from polarization curves (Fig. 9i) and the increasing diameter of capacitive loops (Fig. 9k) with increasing immersing time.

**Figure 11:**
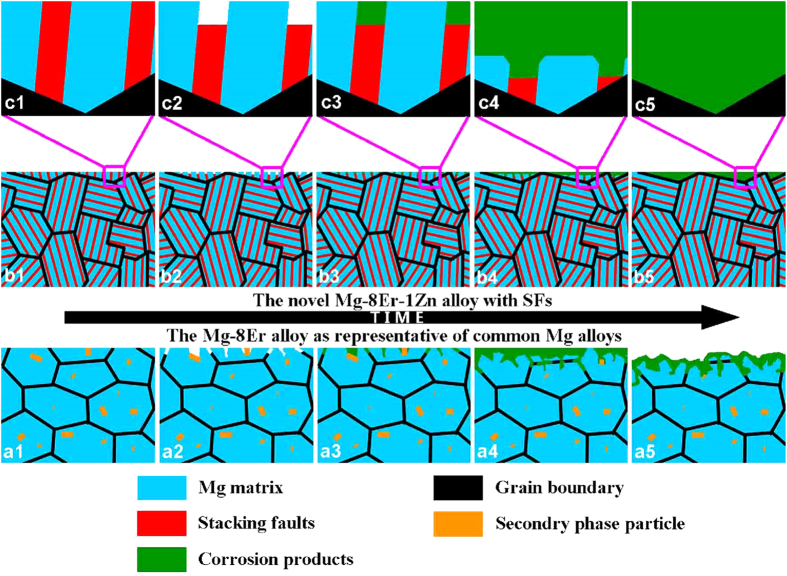
Schematic diagrams of morphology during corrosion process. a1-a5 graphs correspond to the common Mg alloys represented by Mg–8Er alloy in this work; b1-b5 graphs correspond to the new Mg–8Er–1Zn alloy with profuse SFs; c1-c5 graphs represent the local amplification of b1-b5.

Specifically, the schematic diagrams are made to simulate the difference corrosion processes of the Mg–8Er alloy with common microstructure and the Mg–8Er–1Zn alloy with novel SFs, as shown in Fig. 11. In Mg–8Er alloy, micro-galvanic coupling would be built up between the cathodic secondary phase particles and the anodic Mg matrix due to their different standard electrode potentials, which can cause severe pitting corrosion. This is the commonly corrosion phenomenon that exists in normal Mg alloys with intermetallic particles as well as impurity elements. However, the corrosion is fundamentally different in Mg–8Er–1Zn alloy with SFs. SFs are the planar crystal lattice defects with the atomic staggered arrangement, thus it is considered that SFs are the unstable structure compared with Mg matrix with perfect crystal structure in thermodynamics. Therefore, the SFs tend to be corroded in preference to Mg matrix. This trend forces the Mg–8Er–1Zn alloy to corrode preferentially along SFs, and meanwhile the corrosion propagation is difficult to extend across grain boundary due to the different orientation of SFs in different grains. Finally the corroded surface forms the “ditch mode” (clearly seen in Fig. 9d). It should be noticed that the corrosion occurs along the SFs in nano scale, thus the “ditch mode” is a uniform mode in fact. On the other hand, it is also not to be neglected that the quick and effective formation of corrosion film along with the corrosion of SFs (Fig. 9c), which further inhibits the corrosion rate.

In the present study, we developed a novel Mg alloy with profuse nano-spaced basal plane SFs for biodegradable implant applications. This alloy with composition of Mg–8Er–1Zn (wt.%) was fabricated by combined processes of direct-chill semi-continuous casting, heat-treatment and hot-extrusion. Benefiting from SFs formation, the new degradable Mg-based alloy exhibited outstanding integrated performance coupled with acceptable biotoxicity, including high strength, ductility, corrosion resistance and most importantly, the uniquely uniform corrosion mode. According to the observation of corrosion process, an original uniform corrosion mechanism was proposed based on SFs imperfect structure, nano scale, special orientation/distribution and the quickly effective film formation.

## Methods

### Material preparation

To prepare the investigated alloy with preliminary designed composition of Mg–8Er–1Zn (wt.%), pure Mg (99.98 wt.%), Zn (99.95 wt.%) and Mg–20Er (wt.%) master alloy were melted in an electric resistant furnace under protection of Ar atmosphere. The ingot was obtained by semi-continuous casting method. The direct-chill semi-continuous casting process was carried out while the temperature of the melt was ~710 °C, and the casting speed was 100 mm/min. The diameter and length of the final ingots were 100 mm and ~1200 mm, respectively. After heat treatment at 500 °C for 12 h, the ingots were extruded with an extrusion ratio of ~28. The extrusion temperature and extrusion ram speed was about 420 °C and 0.8 mm/s, respectively. The pure Mg and Mg–8Er (wt.%) extruded rods were also prepared by the same method for comparison. Inductively coupled plasma atomic emission spectroscope (ICP-AES) was used to determine the actual chemical compositions of the alloys, and the results were listed in Table 2.

### Electrochemical measurements

Electrochemical measurements were carried out using an AUTOLAB PGSTAT302N electrochemical workstation. A standard three-electrode setup was used to perform electrochemical measurement: i.e. a test sample, a platinum wire and a saturated calomel electrode act as working electrode, counter electrode and reference electrode, respectively. The scan rate of the polarization curves measurement was 2 mV/s. The polarization started from a cathodic potential of −50 mV relative to the open circuit potential and stopped at an anodic potential about −1.4 V. The electrochemical impedance spectroscope (EIS) was carried out at open potential with an applied signal of 10 mV rms. The scanning frequency ranged from 100 kHz down to 10 mHz. Simulated body fluid (SBF) was used in both electrochemical measurements and immersion tests, and the composition was listed in Table 3. Before the test, the specimens were ground successively with SiC abrasive papers from 320 grit to 3000 grit and then cleaned with acetone and deionized water. Every specimen was immersed for 10 min to keep the data at a stable level.

### *In vitro* corrosion (degradation) tests

Immersion tests in SBF were carried out to study the *in vitro* corrosion behavior of the alloys, on the basis of ISO/FDIS 23317:2007(E). The temperature of SBF was maintained at 37 ± 0.5 °C using water bath. The specimens of 12 mm × 12 mm × 5 mm were polished with SiC abrasive papers. Before the tests, the specimens were cleaned with acetone and deionized water using ultrasonic cleaning. The corrosion products on the specimens were cleaned after immersion test by dipping in a solution of 200 g/L CrO_3_, 10 g/L AgNO_3_ and 20 g/L Ba(NO_3_)_2_, according to ISO 8407. At least three samples were tested for each condition. The corroded surface and corrosion film were characterized by scanning electron microscopy (SEM) with energy dispersion spectrometry (EDS) and X-ray photoelectron spectroscope (XPS).

Corrosion rates were obtained by using all the three measuring methods, *P*_w_, *P*_H_ and *P*_i_, respectively. *P*_w_ is the corrosion rate calculated from weight loss for 10 days immersion in SBF; *P*_H_ is the corrosion rate measured from hydrogen evolution for 10 days immersion in SBF; *P*_i_ is the corrosion rate evaluated by Tafel extrapolation from polarization curves measured after 10 min immersion in SBF.


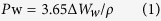



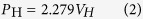



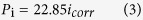



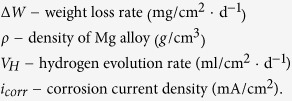


### Biotoxicity test

Roswell Park Memorial Institute-1640 (RPMI-1640) was employed to culture L-929 cells which were used in biotoxicity test. The culture medium was kept at 37 °C in a humidified atmosphere of 5% CO_2_. The cytotoxicity tests were performed in accordance with indirect contact. Extracting solution was prepared according to ISO 10993–5:2009. After 24 h incubation under aforementioned condition, the extraction medium was serially diluted to100%, 75%, 50% and 25% concentrations. As the control groups, pure RPMI-1640 medium served as negative control group and 0.64% phenol RPMI-1640 medium served as positive control group. Incubation of L-929 cells was carried out in 96-well flat-bottomed cell culture plates and 1 × 10^4^ cells/100 μl medium was injected in each well. After first 24 h incubation period for attachment, the medium in well was cleaned and replaced with 100 ml of extracts. And after second 24 h incubation period, 10 μl MTT was added to each well. When the third 4 h incubation period ended, 100 μl formazan solution was added into each well and 100 ml of the supernatant was measured spectrophotometrically at 570 nm by Elx800 (BioTek instruments). The cell viability was calculated according to the following formula:


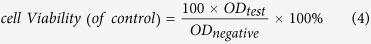



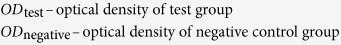


### *In vivo* corrosion tests

**Figure 12:**
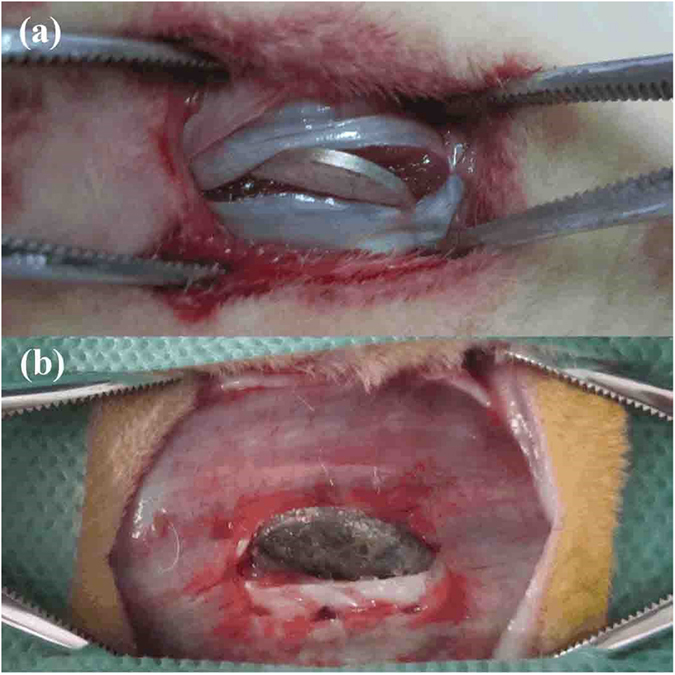
Samples in the erector spinae of rabbit. (**a**) Before and (**b**) after *in vivo* corrosion for 10 days.

3 groups of rabbits were adopted as parallel test. Each groups contained 3 adult male New Zealand rabbits and all rabbits were randomly chosen in the animal laboratory of Harbin Medical University. Each rabbit was implanted with two Mg–8Er–1Zn alloy samples with 15 mm diameter ×1 mm thickness. Animals were generally anesthetized for surgery and the dorsum region was scrubbed with 25 g/L tincture of iodine and 70% ethanol. A pair of samples was implanted into the rabbit’s left and right erector spinae, respectively. After operation, all animals were given a subcutaneous injection of penicillin. Each rabbit was sacrificed 10 days after surgery. Samples in the erector spinae of rabbit before and after *in vivo* corrosion are shown as Fig. 12. All procedures involving animals were approved by Harbin Medical University Ethics Committee for Animal Experiments and performed in accordance with the Guide for the Care and Use of Laboratory Animals published by the US National Institutes of Health. Finally, the corroded surfaces of samples were characterized by SEM with EDS.

### Mechanical property tests

Tensile and compression tests were carried out using an Instron–5569 material testing machine under an initial strain rate of 1 × 10^−3^/s. The tension and compression tests referred to ASTM: E8 / E8M–13a and ASTM: E9–09, respectively. The samples were machined by Electrical Discharge Machining (EDM) and then polished with 800 grit SiC abrasive papers. The length direction of specimens was parallel to the extrusion direction. Every test was repeated for at least five times.

### Microstructure characterization

The microstructure, phase composition and structure of the alloys were characterized by SEM equipped with EDS and electron back-scattered diffraction (EBSD) system, X-ray diffraction (XRD) and transmission electron microscope (TEM). All the specimens for SEM observation were polished with 3000 grit SiC abrasive papers and etched in a 3% HNO_3_/C_2_H_5_OH solution. For EBSD tests, the specimens with size of 4 × 4 × 3 mm^3^ were polished with 3000 grit SiC abrasive papers, and then electrolytic polishing was conducted at constant current with specimen as anode and stainless as cathode in the electrolyte solution (alcohol anhydrous and phosphate, with the volume ratio 3:5). XRD was carried out on Xpert-Pro diffractometer with Cu Kα radiation in the range from 20° to 80° with a step of 0.02°. The thin foil samples for TEM observation were prepared by polishing small plates to thickness of ~30 μm, and finally using Gatan Precision Ion Polishing System until a hole appeared.

## Additional Information

**How to cite this article**: Zhang, J. *et al.* New horizon for high performance Mg-based biomaterial with uniform degradation behavior: Formation of stacking faults. *Sci. Rep.* **5**, 13933; doi: 10.1038/srep13933 (2015).

## Figures and Tables

**Table 1:** Measurements related to corrosion rate in SBF at 37 °C.

Alloy	*ΔW*(mg/cm^2^·d^−1^)	*P*_W_(mm/y)	*V*_H_(ml/cm^2^·d^−1^)	*P*_H_(mm/y)	*i*_corr_ (mA/cm^2^)	*P*_i_ (mm/y)
Mg-8Er	0.852	1.79	0.748	1.70	0.223	5.10
Mg-8Er-1Zn	0.161	0.34	0.121	0.28	0.075	1.71

V_H_ and ΔW were measured using 240 h immersion. Tafel extrapolation evaluated *i*_*corr*_ from polarization curves measured after 10 min immersion.

**Table 2:** Actual chemical compositions of alloys.

Alloy	Er (wt.%)	Zn (wt.%)	Fe (wt.%)	Ni (wt.%)	Cu (wt.%)	Mg (wt.%)
Mg–8Er	8.244	/	0.003	0.002	0.004	Bal.
Mg–8Er–1Zn	8.167	1.125	0.004	0.003	0.004	Bal.

**Table 3:** Detailed composition of the SBF solution.

Component	Mass (g/l)
NaCl	8.035
NaHCO_3_	0.355
KCl	0.225
K_2_HPO_4_·3H_2_O	0.231
MgCl_2_·6H_2_O	0.311
c(HCl) = 1 mol/l	39 ml
CaCl_2_	0.292
Na_2_SO_4_	0.072
TRIS	6.118
